# A Novel Bacteriocin From *Lactobacillus Pentosus* ZFM94 and Its Antibacterial Mode of Action

**DOI:** 10.3389/fnut.2021.710862

**Published:** 2021-07-23

**Authors:** Mengdi Dai, Yanran Li, Luyao Xu, Danli Wu, Qingqing Zhou, Ping Li, Qing Gu

**Affiliations:** Key Laboratory for Food Microbial Technology of Zhejiang Province, College of Food Science and Biotechnology, Zhejiang Gongshang University, Hangzhou, China

**Keywords:** bacteriocin, purification, pentocin ZFM94, antibacterial activity, mode of action

## Abstract

Bacteriocins are bioactive antimicrobial peptides synthesized in the ribosome of numerous bacteria and released extracellularly. Pentocin ZFM94 produced by *Lactobacillus pentosus (L. pentosus)* ZFM94, isolated from infant feces with strong antibacterial activity, was purified by ammonium sulfate precipitation, dextran gel chromatography, and reverse-phase high-performance liquid chromatography (RP-HPLC). The molecular mass of the purified bacteriocin was 3,547.74 Da determined by matrix-assisted laser desorption/ionization time-of-flight mass spectrometry (MALDI-TOF MS). Pentocin ZFM94 exhibited broad-spectrum antimicrobial activity against tested Gram-positive and Gram-negative bacteria, and the minimal inhibitory concentrations (MICs) of *Micrococcus luteus (M. luteus)* 10,209, *Staphylococcus aureus* (*S. aureus*) D48, and *Escherichia coli (E. coli)* DH5α were 1.75, 2.00, and 2.50 μm, respectively. Pentocin ZFM94 was heat-stable (30 min at 80°C) and showed inhibitory activity over a wide pH range (5.00–7.00). It could be degraded by trypsin and pepsin, but not by amylase, lysozyme, lipase, and ribonuclease A. Fluorescence leakage assay showed that pentocin ZFM94 induced disruption of the cell membrane and caused leakage of cellular content. Furthermore, lipid II was not an antibacterial target of pentocin ZFM94. This study laid the foundation for further development and utilization of *L. pentosus* ZFM94 and its bacteriocin.

## Introduction

Bacteriocins are ribosomally synthesized peptides that in most cases they exhibit antibacterial activity against bacteria that are closely related to the producing bacteria. As for food corruption, the commonly used method is to add preservatives. Although, the toxicity of the preservatives now used is low, the toxicity accumulates and damages the body when taken in excess. Non-toxic natural preservatives solve this problem perfectly. The bacteriocins are of very high perspective because they may not only be used for food biopreservation but also have the potential to be utilized as antibiotics, exploited in animal healthcare and marine environment (1). Bacteriocins are usually divided into four groups (2). Class I bacteriocins, known as lantibiotic, generally have a molecular weight of <5 kDa (3), such as lanthionine, β-12 methyllanthionine, dehydrobutyrine, dehydroalanine, and labyrinthine. Class I is further subdivided into class Ia (lantibiotics), class Ib (labyrinthopeptins), and class Ic (sanctibiotics). Nisin is a most popular class I bacteriocin (4). Class II bacteriocins are called non-lantibiotics with molecular weight <10 kDa (5), which contain 30–60 amino acids with thermal stability, and Class II bacteriocins can be further subdivided into class IIa (pediocin-like bacteriocins), class IIb (two-peptide unmodified bacteriocins), class IIc (circular bacteriocins), and class IId (unmodified, linear, and non-pediocin-like bacteriocins). Pediocin-like bacteriocins are the most dominant class IIa bacteriocins (4). Class III bacteriocins are non-lantibiotics with heat sensitivity, and their molecular weight is generally >30 kDa (6). Colicin is one of the examples of class III bacteriocins produced by *Escherichia coli*. Class III bacteriocins also include helveticin M, helveticin J, and enterolysin A produced by *Lactobacillus crispatus* and *Lactobacillus helveticus*, respectively (4). Class IV bacteriocins are comprised of lipid or carbohydrate groups (7). Besides, many bacteriocin-producing strains have been found, such as *Streptococcus lactis, Listeria monocytogenes (L. monocytogenes)*, and *Staphylococcus aureus (S. aureus)* (4).

Lactic acid bacteria (LAB) are food-grade microorganisms recognized by the Food and Drug Administration (FDA). Recently, the isolation of bacteriocins has become a research hotspot. The most well-known bacteriocin produced by LAB is nisin (8). It is an FDA-approved and generally regarded as safe (GRAS) peptide with recognized potential for food preservation, which has been commercialized in more than 50 countries (9). Nisin was effective in the treatment of dental caries and ulcers in humans, and mastitis in cattle (8, 10).

Studies on the mode of action and target of bacteriocins can lay a foundation for its applications. The action mode of bacteriocins can generally be grouped into two types. The first one is acting on the cell membrane of the bacteria, which can be further divided into two subclasses: targeting cell membrane protein (11) and non-specific “membrane perforation.” Nisin can inhibit the growth of Gram-positive bacteria through two antibacterial mechanisms. Lipid II, as a membrane-anchored cell wall precursor, is essential for the biosynthesis of the bacterial cell wall. When nisin binds with lipid II, the biosynthesis of the cell wall is inhibited, resulting in the death of the bacteria (12). Nisin can also cause the loss of proton motive force (PMF) of the cell membrane through the formation of non-selective transmembrane pores at micromolar concentrations (13). The second type is directed to the destruction of non-membrane substances in cells, for example, the target of microcin B17 is DNA gyrase (14).

Although, bacteriocinogenic *Lactobacillus Pentosus (L. pentosus)* has been isolated from different products such as fermented meat and cereals, there are few reports on the purification of bacteriocin from *L. pentosus* (15–19). *Lactobacillus Pentosus* ZFM94 was isolated from the feces of a healthy infant with strong antibacterial activities and probiotic properties (20). In this study, a three-step method was established to purify a novel bacteriocin (pentocin ZFM94) from the cell-free supernatant (CFS) of *L. pentosus* ZFM94. The antibacterial spectrum and minimum inhibitory concentrations (MICs) of pentocin ZFM94 were determined. Meanwhile, the effects of temperature, pH, and enzyme on the stability of pentocin ZFM94 were studied. Finally, the leakage experiment and agar well-diffusion method were used to explore the action mode of pentocin ZFM94 and its binding site with lipid II.

## Materials and Methods

### Strains and Culture Conditions

*Lactobacillus pentosus* ZFM94 was isolated from the feces of a healthy infant, deposited at the China Center for Type Culture Collection (CCTCC) with the strain number of CCTCC NO: M 2016632, and cultured in de Man, Rogosa, and Sharpe (MRS) broth at 37°C. The indicator strains are listed in Table 1.

**Table 1:** Inhibition spectrum of pentocin ZFM94 against different bacterial indicator strains.

**Indicator strains**	**Growth condition**	**Antimicrobial activity**	**Sources**
*Micrococcus luteus* 10209	LB at 30°C	++++	Laboratory preservation
*Staphylococcus aureu*s D48	LB at 37°C	+++	Laboratory preservation
*Staphylococcus carnosus* pCA44	LB at 37°C	++	Laboratory preservation
*Staphylococcus carnosus* pot20	LB at 37°C	+	Laboratory preservation
*Listeria monocytogenes* LM1	LB at 37°C	+	Laboratory preservation
*Bacillus subtilis* BAS2	LB at 37°C	–	Laboratory preservation
*Escherichia coli* DH5α	LB at 37°C	+++	Laboratory preservation
*Salmonella paratyphi-A* CMCC50093	LB at 37°C	–	National Center for Medical Culture Collections (CMCC)
*Salmonella paratyphi-B* CMCC50094	LB at 37°C	–	National Center for Medical Culture Collections (CMCC)
*Salmonella enterica* subsp. enterica ATCC14028	LB at 37°C	+	American Type Culture Collection (ATCC)
*Saccharomyces cerevisiae* SM190	YPD at 28°C	+	Laboratory preservation

*Inhibitory zone diameter (mm): ++++ 22–25, +++ 18–21, ++ 14–17, + 10–13, – no inhibition*.

### Purification of Bacteriocin

A three-step method, including ammonium sulfate precipitation, Sephadex™ G-25, and reverse-phase high-performance liquid chromatography (RP-HPLC) were established to purify bacteriocin. Four liters of clarified MRS broth was inoculated (1%) with *L. pentosus* ZFM94 and incubated for 18–20 h at 37°C. The culture was centrifuged (Beckman, USA) at 8,000 rpm and 4°C for 20 min. The CFS was precipitated with saturated ammonium sulfate (gradient from 10 to 90%). The protein precipitations were collected by centrifugation (8,000 rpm, 4°C, 20 min), dissolved in 50 mm phosphate buffer saline (PBS, pH 3.69), and desalted by dialysis (1 kDa cutoff membrane, Sangon, China). The active fractions were purified by Sephadex G-25 (GE Healthcare, 1.6 × 80 cm) equilibrated and eluted with ultrapure water at 1 ml/min. The eluent was collected every 3 min until no absorbance was detected at 280 nm. Next, the active ingredient was concentrated by a vacuum rotary evaporator (Marin Christ, Germany). Subsequently, it was introduced into the RP-HPLC system (Waters, USA) equipped with a RP C18 column (YMC-Pack ODS-AQ, 150 × 20 mm L.D.). The buffer A was 0.05% trifluoroacetic acid (TFA)/distilled water (v/v), and buffer B was 0.05% TFA/acetonitrile (v/v). Gradient elution ranged from 95% buffer A to 95% buffer B, with a flow rate of 4.0 ml/min. Fractions with antibacterial activity were analyzed by an analytical RP-HPLC system (Waters 2489 Detector, USA) using RP C18 column (YMC-Pack ODS-AQ, 150 × 4.6 mm L.D., Japan) after removing acetonitrile by evaporation. Protein concentration was determined by a bicinchoninic acid (BCA) protein assay kit according to the instructions of the manufacturer (Takara, Dalian, China).

### Determination of the Molecular Weight of Pentocin ZFM94

The rough molecular mass of sample fraction with antibacterial activity prepared by RP-HPLC was analyzed by Tricine-sodium dodecyl sulfate–polyacrylamide gel electrophoresis (SDS-PAGE) (5% concentrated glue and 18% separation glue). The antibacterial activity and a low molecular weight marker were run at 80 V for 30 min and 120 V during the rest of the separation. After completion of the run, the gel was stained with Coomassie Brilliant Blue G-250 and destained by ethyl alcohol acetic acid solution. Matrix-assisted laser desorption/ionization time-of-flight mass spectrometry (MALDI-TOF MS) (ABSciex 5800) was further used to determine the accurate molecular weight of this sample (21), which was operated in positive ion mode. The α-cyano-4-hydroxy-cinnamic acid (CHCA) solution was used as the matrix. In brief, 1 μl of the sample was dried on the sample target plate, and then 0.6 μl of CHCA was added after natural drying for the MALDI analysis.

### Antibacterial Spectrum and MICs of Pentocin ZFM94

The antibacterial spectrum of pentocin ZFM94 after purification was detected by the agar well-diffusion method (22). Soft agar of each medium was inoculated with 10^6^ colony-forming units (CFU)/ml of each indicator strain (Table 1) and mixed well, and 8 mm diameter wells were punched with Oxford Cups in the plates. Every well was filled with 100 μl of 10 μm pentocin ZFM94, and the plates were incubated overnight at 37°C. The diameter of the inhibition zones (mm) around the wells was measured.

According to the results of the antibacterial spectrum, MICs of pentocin ZFM94 on *S. aureus* D48, *Micrococcus luteus* 10209, and *E. coli* DH5α were determined by tube method (23). The concentration gradient was 0, 0.10, 0.20, 0.50, 0.875, 1.75, 2.00, 2.50, 3.00, and 10.00 μm. The absorbance was measured by an ultraviolet spectrophotometer (OLYMPUS, Japan). Each concentration was carried out in triplicate.

### Stability of Pentocin ZFM94 on pH, Heat, and Enzyme Conditions

Purified pentocin ZFM94 was prepared at a concentration of 15 μg/m and was used for testing the effects of heat, pH, and enzymes. To determine thermal stability, the purified pentocin ZFM94 was heated to 50, 60, 70, 80, 90, 100, and 121°C for 30 min, respectively, and then cooled to room temperature. To evaluate the susceptibility of pentocin ZFM94 to different pH values, the pH of purified peptide solution was adjusted to 2–10 using 1 M hydrochloric acid (HCl) and 1 M sodium hydroxide (NaOH), respectively. The above samples were then incubated at 4°C for 2 h and adjusted to an initial pH of 3.69. For proteolytic enzyme treatments, pentocin ZFM94 was treated with lysozyme, ribonuclease A, lipase, papain, α-amylase, α-chymotrypsin, pepsin, and trypsin at the final concentration of 1 mg/ml, under the optimal temperature and pH of each enzyme for 2 h, and then, the pH was adjusted to initial pH of 3.69. The activity of residual anti-*M. luteus* 10209 was calculated by the agar well-diffusion test. An untreated peptide sample was taken as a control.

### Analysis of Transmembrane Electrical Potential

Fluorescence leakage test was used to measure transmembrane electrical potential (ΔΨ) (24), and the response value of the cell membrane probe [3, 3-dipropylthiadicarbocyanine iodide, DisC2(5)] was measured by a fluorescence spectrophotometer (Agilent, USA). Luria-Bertani (LB) broth (20 ml) was inoculated with a 1% overnight culture of *M. luteus* 10209. The inoculated broth was grown until it reached the exponential phase of growth (OD_600_ = 0.6–0.8). Cells were collected by centrifugation at 4,000 rpm and 4°C for 30 min, washed twice with buffer (250 mm glucose, 5 mm magnesium sulfate (MgSO_4_), 10 mm tripotassium phosphate (K_3_PO_4_), and 100 mm potassium chloride (KCl), pH 7.0), and dissolved in 2 ml of the same buffer, by adding 20 μl strain and 2 μl DisC2(5) into 2 ml buffer in order. When the detected fluorescence value was stable, pentocin ZFM94 with final concentrations of 1.75 and 8.75 μm was added, respectively. The same volume of 0.05% acetic acid was used as a negative control.

### Study on the Binding of Pentocin ZFM94 to Lipid II

Studies have shown that lipid II was the target of nisin (25), and the combination of lipid II and nisin can mediate the formation of pores. Nisin and pentocin ZFM94 were mixed with lipid II, respectively. The final concentration of nisin or pentocin ZFM94 was 10 μm, while the concentration of lipid II was 20 μm. The mixture was maintained at 4°C for 1 h. The changes in the antibacterial activity were detected by the agar well-diffusion method. *Micrococcus luteus* 10209 was used as the indicator. Then, the size of the inhibition zone was observed.

## Results

### Purification of Bacteriocin

**Figure 1:**
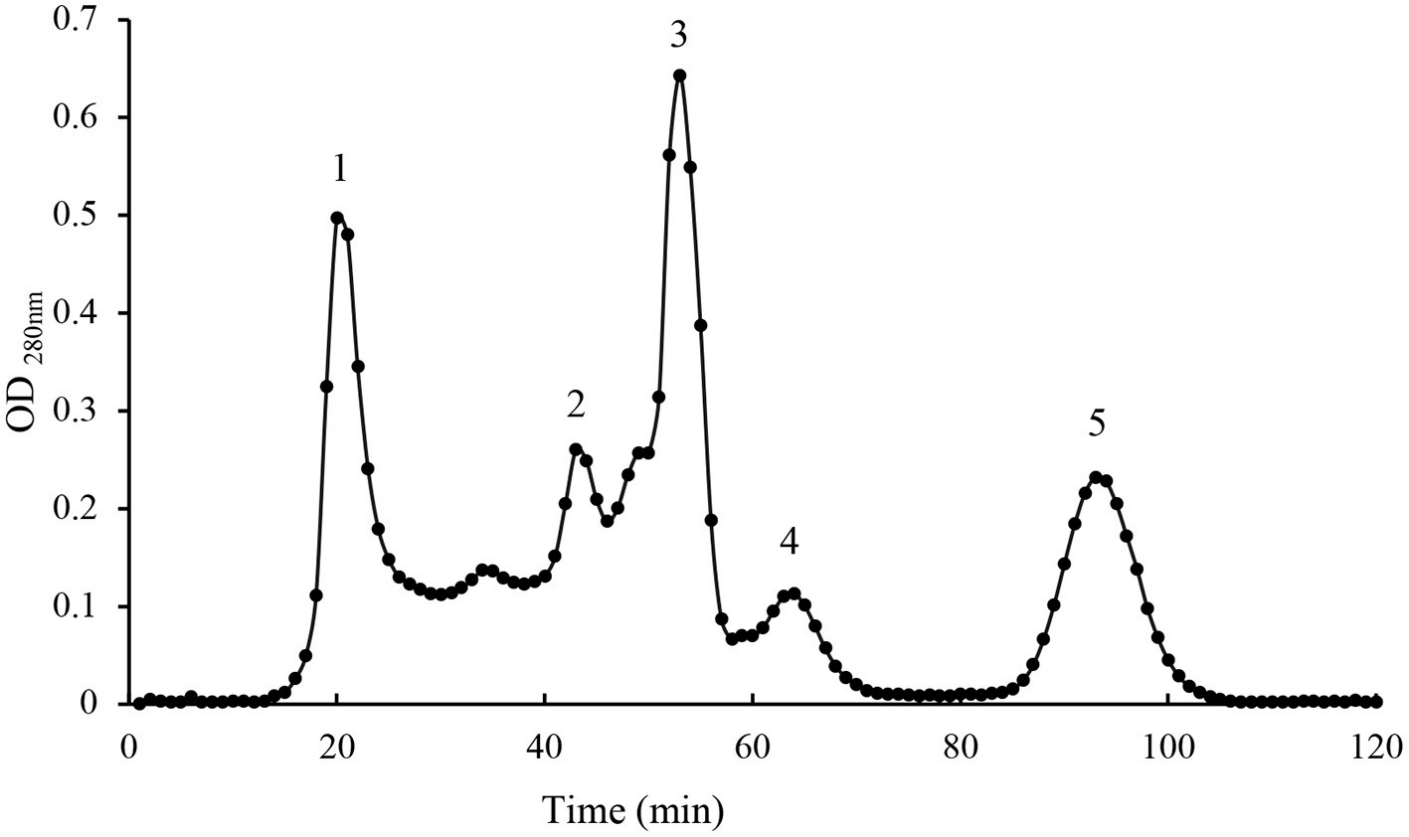
Gel filtration chromatography peak of active fractions.

Pentocin ZFM94 was purified from a 4 L culture supernatant. The obtained precipitate had good antibacterial activity when the saturation of ammonium sulfate was 40%. Then, Sephadex G-25 was used to separate and acquire the range between 1 and 5 kDa. Ultrapure water was used as a buffer. When the sample was added to the dextran gel chromatography, it was separated according to the molecular weight. About 40% ammonium sulfate precipitation component can be well-separated from Sephadex G-25. Five peaks were obtained as shown in Figure 1. Only peak 3 had antibacterial activity against *M. luteus*10209.

**Figure 2:**
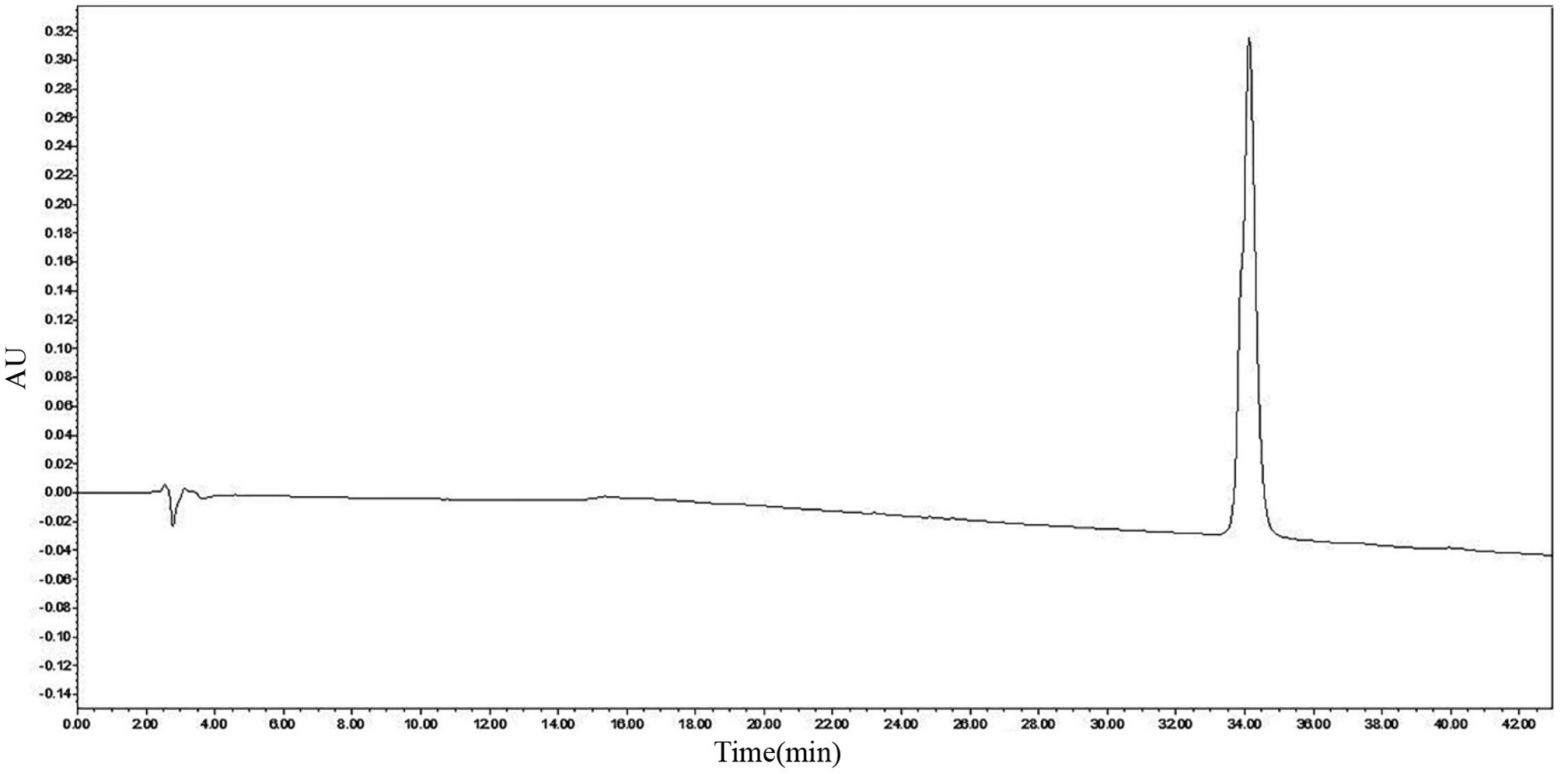
Reverse-phase high-performance liquid chromatography (RP-HPLC) analysis of pentocin ZFM94.

After purification by Sephadex G-25, RP-HPLC was used to purify the active ingredient. The active ingredient was obtained at a retention time of 22–23 min using the preparative C18 column. Analytical column C18 was used to analyze bacteriocin, and only a single peak at 34.129 min was observed, as shown in Figure 2. The purity of bacteriocin obtained by the area normalization method was 98.70%. The concentration of bacteriocin was 3.65 mg/L.

### Molecular Weight Determination of Purified Bacteriocin

**Figure 3:**
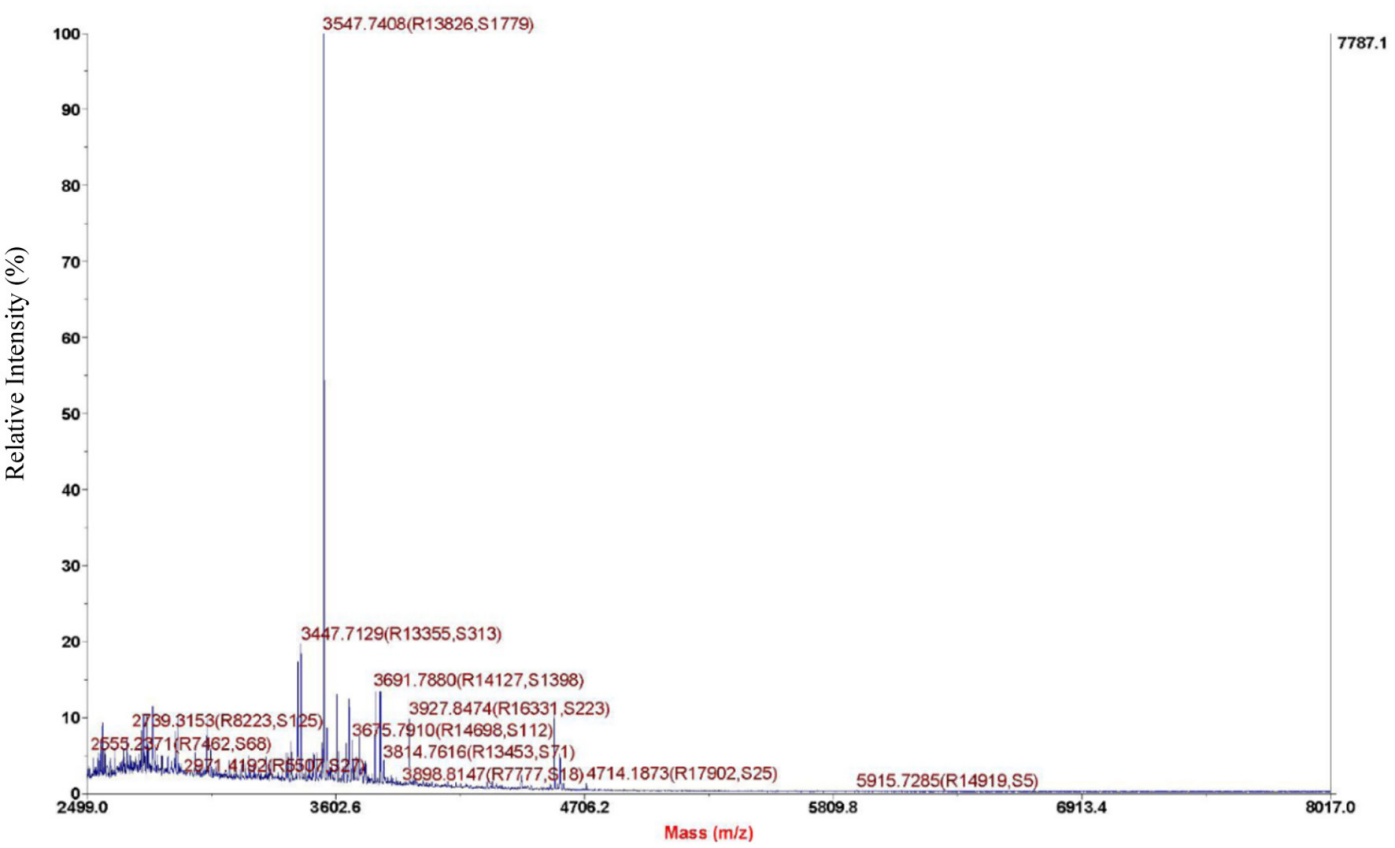
Molecule weight of pentocin ZFM94 determined by matrix-assisted laser desorption/ionization time-of-flight mass spectrometry (MALDI-TOF MS).

As we can see from the result of Sephadex G-25, the molecular weight of purified bacteriocin was <5 kDa. Tricine-SDS-PAGE analysis was performed with purified bacteriocin. The molecular mass determined by relative mobility was between 1.7 and 4.6 kDa. Purified bacteriocin had an accurate molecular mass of 3,547.74 Da by MALDI-TOF MS as shown in Figure 3 and named pentocin ZFM94.

### Antimicrobial Spectrum and MICs

Pentocin ZFM94 showed the inhibitory effects against most Gram-positive bacteria, some Gram-negative bacteria, and fungi, as shown in Table 1. Among the indicator species, pentocin ZFM94 showed high activities against *M. luteus* 10209, *S. aureus* D48, and *E. coli* DH5α. In addition, it inhibited *L. monocytogenes* LM1, *Salmonella enterica subsp. enterica* ATCC14028, and *Saccharomyces cerevisiae* SM190. However, pentocin ZFM94 had no inhibitory activity against *Bacillus subtilis* BAS2, *Salmonella paratyphi-B* CMCC50094, and *Salmonella paratyphi-A* CMCC50093. Pentocin ZFM94 also inhibited Gram-positive bacteria *S. carnosus* pot20 and *L. monocytogenes* LM1, and *Saccharomyces cerevisiae* SM190. These results demonstrated that this bacteriocin had a broad antibacterial activity.

Minimal inhibitory concentrations were determined by the test tube method (23). Values of MICs for *M. luteus* 10209, *S. aureus* D48, and *E. coli* DH5α were 1.75, 2.00, and 2.50 μm, respectively, which exhibited obvious antibacterial activities.

### The Effects of pH, Temperature, and Enzymes on Pentocin ZFM94

**Figure 4:**
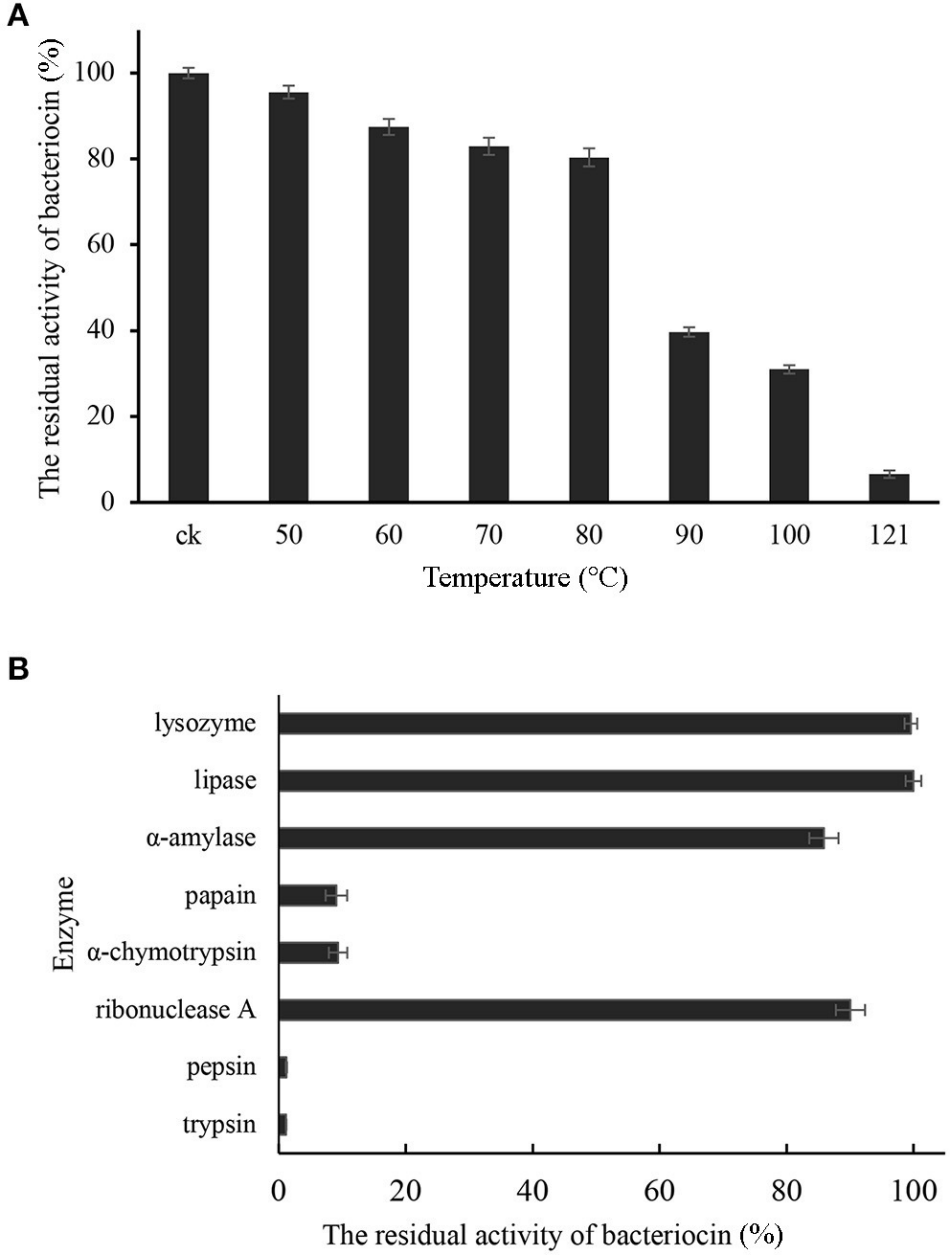
**(A)** Effect of temperature on the antibacterial activity of pentocin ZFM94; **(B)** Stability of pentocin ZFM94 after different enzyme treatments.

Pentocin ZFM94 had the best bacteriostatic effect at pH 3 and pH 6; when the pH increased from 8 to 10, the inhibition activity was significantly declined. Pentocin ZFM94 maintained complete activity after exposure from 50 to 80°C for 30 min (Figure 4A). When the temperature was higher than 80°C, the inhibition activity was significantly descended. The effects of enzymes were presented in Figure 4B. The activity of pentocin ZFM94 was significantly reduced by treatment with pepsin and trypsin. However, amylase, lysozyme, lipase, and ribonuclease A did not effect on the antimicrobial activity.

### Antibacterial Action Mode of Pentocin ZFM94

**Figure 5:**
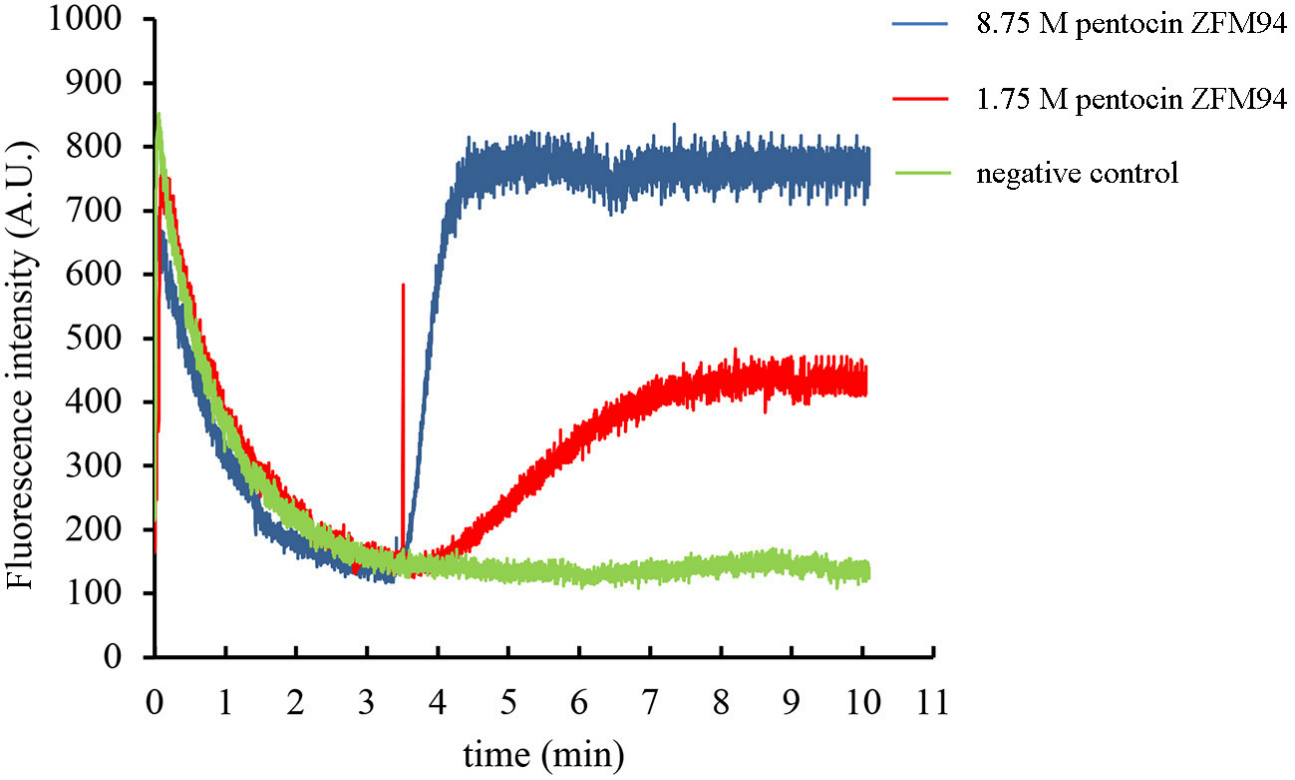
Effect of different pentocin ZFM94 concentrations on the membrane of *Micrococcus luteus* (*M. luteus*).

The effect of pentocin ZFM94 on the membrane integrity of *M. luteus* 10209 was determined by the membrane potential sensitive dye DisC2(5). After the addition of DisC2(5), the fluorescence value decreased and reached stability in 3 min. When 0.05% acetic acid was added, the fluorescence value did not change. As shown in Figure 5, with the addition of pentocin ZFM94 at a final concentration of 8.75 μm (5 × MIC), the fluorescence value increased faster than the final concentration of 1.75 (1 × MIC). It can be seen that the antibacterial action of pentocin ZFM94 was through the perforation of the bacterial membrane, and indicator bacteria were killed by causing leakage of intracellular electrolytes.

**Figure 6:**
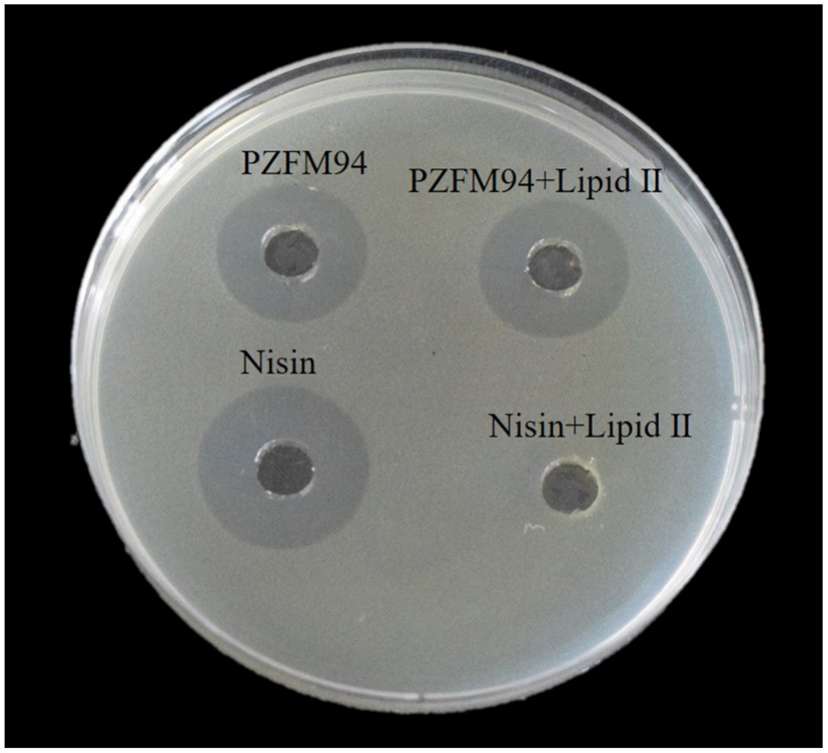
Binding experiment of pentocin ZFM94 with lipid II.

In order to further explore whether lipid II was the target of pentocin ZFM94, nisin was used as a control and *M. luteus*10209 was used as an indicator. The lipid II (20 μm) was mixed at a ratio of 2:1 with the pentocin ZFM94 (10 μm). The bacteriostatic activity was detected by the agar well-diffusion method as shown in Figure 6. After pentocin ZFM94 was mixed with lipid II, the inhibitory action of penticin ZFM94 did not change, but the antibacterial effect of nisin disappeared. The result showed that lipid II was not the target of pentocin ZFM94.

## Discussion

Bacteriocins are generally defined as a class of small molecular proteins or peptides synthesized by ribosomes in the metabolic process of some bacteria. *Lactobacillus Pentosus* has antibacterial activity, which is mainly related to its metabolites (26), and bacteriocin is one of them. *Lactobacillus Pentosus* can produce bacteriocin that has been reported, pentocin MQ1 produced by *L. pentosus* CS2 (19), and pentocin TV35b produced by *L. pentosus* TV35b (27). The appropriate separation and purification processes vary from different bacteriocins. Pentocin TV35b was purified by ammonium sulfate precipitation, followed by sulfopropyl (SP)-sepharose cation exchange chromatography (27). Pentocin JL-1 was purified using macroporous resin, cation exchange, gel filtration, and semipreparative HPLC (17). Pentocin MQ1 was purified using the adsorption-desorption approach followed by RP-HPLC (19). In our study, pentocin ZFM94 was purified by a three-step process, including ammonium sulfate saturation at 40%, Sephadex G-25, and RP-HPLC from the supernatant of *L. pentosus* ZFM94. Then, we determined its molecular weight, determined its antibacterial spectrum, and explored its antibacterial mode.

Pentocin ZFM94 exhibited high thermal and pH stability. It retained 80.37% of its original antibacterial activity after heating at 80°C for 30 min. The antibacterial activity of pentocin ZFM94 was stable at pH 2–7. Pentocin MQ1 retained activity at 40–121°C, but unlike pentocin ZFM94, it was inactive at pH 6–7 (19). Nisin also exhibited a strong antibacterial activity at low pH, but it will be inactivated at pH close to 7 (28). Pentocin ZFM94 was sensitive to trypsin and pepsin, but not to amylase, lysozyme, lipase, and ribonuclease A. Studies have reported that leuconocin S and carnocin 54 are sensitive to amylase but are not sensitive to protease (29). Thus, pentocin ZFM94 has a proteinaceous nature like most other bacteriocins.

Pentocin ZFM94 performed activity against Gram-positive bacteria, Gram-negative bacteria, and fungi, especially for *S. aureus* D48, *M. luteus* 10209, and *E. coli* DH5α. Many pentocins produced from *L. pentosus* also can inhibit a variety of Gram-positive bacteria, Gram-negative bacteria, and the fungi *Candida albicans* (27). Food spoilage caused by microbial contamination has been a huge challenge for production for the food industry. But so far, only nisin was allowed to be used as a food preservative. Nisin only can inhibit Gram-positive bacteria, and plantaricin 163 can inhibit Gram-positive and Gram-negative bacteria but cannot inhibit fungi (30). Most bacteriocins could not be widely and effectively used in the food industry due to their narrow spectrum and exhibit inhibitory activity against only genetically close bacteria. Therefore, pentocin ZFM94 has a wider application prospect. The MICs of pentocin ZFM94 against *S. aureus* D48, *M. luteus* 10209, and *E. coli* DH5α were 2.00, 1.75, and 2.50 μm. BMA has a molecular weight of 1.77 kDa produced by *L. crustorum* MN047, and MICs toward *S. aureus* ATCC 29213 and *E. coli* ATCC 25922 were 165 and 305 μm, respectively (21). Pentocin ZFM94 performed a higher activity against *S. aureus* and *E. coli* than bacteriocin BMA.

The mode of action of many bacteriocins has been identified to inhibit microorganisms by disrupting the cell membrane integrity of microorganisms (31, 32). In our study, 1 × and 5 × MIC of pentocin ZFM94 were tested using a fluorescence leakage test. Both concentrations of pentocin ZFM94 were able to disrupt the integrity of *M. luteus* 10209 cell membrane. In addition, the disruption of cell membrane integrity was dose dependent. Similar dose-dependent inhibitory action has also been observed by aureocin A53 (32) and Plantaricin EF (Pln EF) (21). However, whether it is a specific membrane perforation still needs further study. Pediocin PA-1 (33) and Lactococcin G (34) all have specific membrane perforation mechanisms.

The mode of action of bacteriocin is not unique. Nisin not only has specific membrane perforation but also can mediate the formation of the hole by combining with lipid II. Studies also found that lipid II was the target of many bacteriocins, such as nisin (12), mersacidin (35), and plantaricin C (36). The molar ratio 1:2 of pentocin ZFM94 and lipid II was studied using the agar well-diffusion test. After pentocin ZFM94 was mixed with lipid II, the inhibitory action did not change suggesting that lipid II is not the target of pentocin ZFM94.

In future studies, we will further optimize the purification method of pentocin ZFM94 and increase the recovery of it. The target of the action, the amino acid sequence, and the structure of pentocin ZFM94 will be addressed in more detail.

## Data Availability Statement

The original contributions generated for the study are included in the article/supplementary material, further inquiries can be directed to the corresponding author/s.

## Author Contributions

QG, PL, and QZ conceived and designed the study. LX, MD, and YL completed the experiment. MD and DW conducted analysis the results and finished the paper. All authors contributed to the article and approved the submitted version.

## Conflict of Interest

The authors declare that the research was conducted in the absence of any commercial or financial relationships that could be construed as a potential conflict of interest.

## Publisher's Note

All claims expressed in this article are solely those of the authors and do not necessarily represent those of their affiliated organizations, or those of the publisher, the editors and the reviewers. Any product that may be evaluated in this article, or claim that may be made by its manufacturer, is not guaranteed or endorsed by the publisher.
